# Protein Folding Modulation in Cells Subject to Differentiation and Stress

**DOI:** 10.3389/fmolb.2019.00038

**Published:** 2019-05-24

**Authors:** David Gnutt, Linda Sistemich, Simon Ebbinghaus

**Affiliations:** ^1^Institute of Physical and Theoretical Chemistry, TU Braunschweig, Braunschweig, Germany; ^2^Department of Physical Chemistry II, Ruhr University Bochum, Bochum, Germany

**Keywords:** cytomimetic media, protein folding and stability, in-cell spectroscopy, Macromolecular crowding, fast relaxation imaging (FReI)

## Abstract

Cytomimetic media are used to mimic the physicochemical properties of the cellular milieu in an *in vitro* experiment. The motivation is that compared to entire cells, they can be used efficiently in combination with a broad range of experimental techniques. However, the development and use of cytomimetic media is hampered by the lack of in-cell data that could be used as a hallmark to directly evaluate and improve the performance of cytomimetic media in different applications. Such data must include the study of specific biomolecular reactions in different cell types, different compartments of a single cells and different cellular conditions. In previous studies, model systems such as cancer cell lines, bacteria or oocytes were used. Here we studied how the environment of cells that undergo neuronal differentiation or proteostasis stress modulates the protein folding equilibrium. We found that NGF induced differentiation leads to a decrease of the melting temperature and a change of the folding mechanism. Proteomic changes that occur upon differentiation could explain this effect, however, we found that the crowding effect remained unchanged. Using MG132, a common proteasome inhibitor and inducer of the unfolded protein response, we show that changes to the quality control machinery modulate the folding equilibrium, leading to protein destabilization at prolonged stress exposure. Our study explores the range of protein folding modulation within cells subject to differentiation or stress that must be encountered in the development of cytomimetic media.

Cytomimetic media allow to reduce the spatial and temporal heterogeneity of compartmentalized cells to homogeneous solutions and allow to study the effect of individual properties of the cellular milieu, like increased viscosity or macromolecular crowding, on a biomolecular reaction. Bridging the gap between the test tube and the cell, they could be used as additives to make *in vitro* experiments, such as high throughput drug screening assays in pharmaceutical industry, more cell-like and more reliable. A hallmark of the cellular milieu is the high density of macromolecules, filling up to 40% of a cell's volume (Zimmerman and Trach, 1991; Rivas and Minton, 2016). Crowding modulates the protein folding equilibrium and should be considered when studying folding in the context of protein misfolding diseases such as Alzheimer's, Parkinson's, Huntington's, and type II diabetes.

In crowded conditions, the protein folding equilibrium is modulated by a combination of excluded volume effects, solvent-mediated effects and so called “soft,” “chemical,” and “quinary” interactions (Zhou et al., 2008; Benton et al., 2012; Wirth and Gruebele, 2013; Monteith and Pielak, 2014; Senske et al., 2014, 2016; Monteith et al., 2015; Gnutt and Ebbinghaus, 2016; Rivas and Minton, 2016). Excluded volume effects stabilize proteins since they favor the more compact folded state compared to the unfolded state. Further, osmolytes and crowders stabilize proteins involving different enthalpic and entropic contributions to the folding free energy. Quinary interactions refer to weak and transient interactions between the protein and the crowder. Depending on the type of protein, the cosolute or solvent conditions, the net effect of these interactions can be either stabilizing or destabilizing.

So far, only a few studies addressed protein folding directly inside the cell. In most cases, model systems such as cancer cell lines or bacteria were utilized to study protein folding via in-cell NMR or fluorescence microscopy (Ignatova and Gierasch, 2004; Ebbinghaus et al., 2010; Danielsson et al., 2015). Such model systems were mostly used without considering the changes of the cellular milieu upon environmental stimuli. One prime example in which crowding was shown to change is osmotic stress and the process of osmotic stress adaptation (Gnutt et al., 2015, 2017). Here we study protein folding in cells undergoing neuronal differentiation and conduct complementary experiments to understand the physicochemical changes of the cellular milieu.

First, we tested if the crowding effect in cells changes upon nerve growth factor (NGF) induced differentiation of PC12 cells. Therefore, we used a Förster resonance energy transfer (FRET) based genetic crowding sensor introduced previously (Boersma et al., 2015), using Clover as FRET donor and mRuby2 as FRET acceptor (Lam et al., 2012), respectively. The sensor detects increases in crowding density by a conformational transition to a more compact state. This leads to a higher FRET efficiency thereby affecting the ratiometric fluorescence readout (higher A/D) (Boersma et al., 2015). The crowding sensor was transiently transfected into HeLa and PC12 cells. Undifferentiated PC12 cells were kept in serum containing growth media, whereas treated PC12 cells were differentiated using NGF and low serum conditions for 7 d. Widefield-fluorescence images show a clear neurite outgrowth in NGF treated cells (Figure S1, Figure 1A). Ratiometric evaluation, however, did not reveal any significant difference between differentiated, non-differentiated PC12 and HeLa cells (Figure 1B). In contrast to HeLa cells, however, the heterogeneity between cells increased which indicated differences between individual differentiated cells. The increase in variance between PC12 and HeLa cells was confirmed by a Bartlett's test for variances (*p* < 0.001). The higher variance observed for PC12 cells could stem from the intrinsic differentiation mechanisms in PC12 cells. While HeLa cells are continuously proliferating, PC12 cells respond to environmental change and undergo differentiation. Therefore, multiple states of the cell could be sampled in the experiments. Further, differentiating PC12 cells are polarized cells and the higher per-cell variability could stem from subcellular changes in crowding that are more homogeneous in HeLa cells and more heterogeneous in PC12 cells.

**Figure 1:**
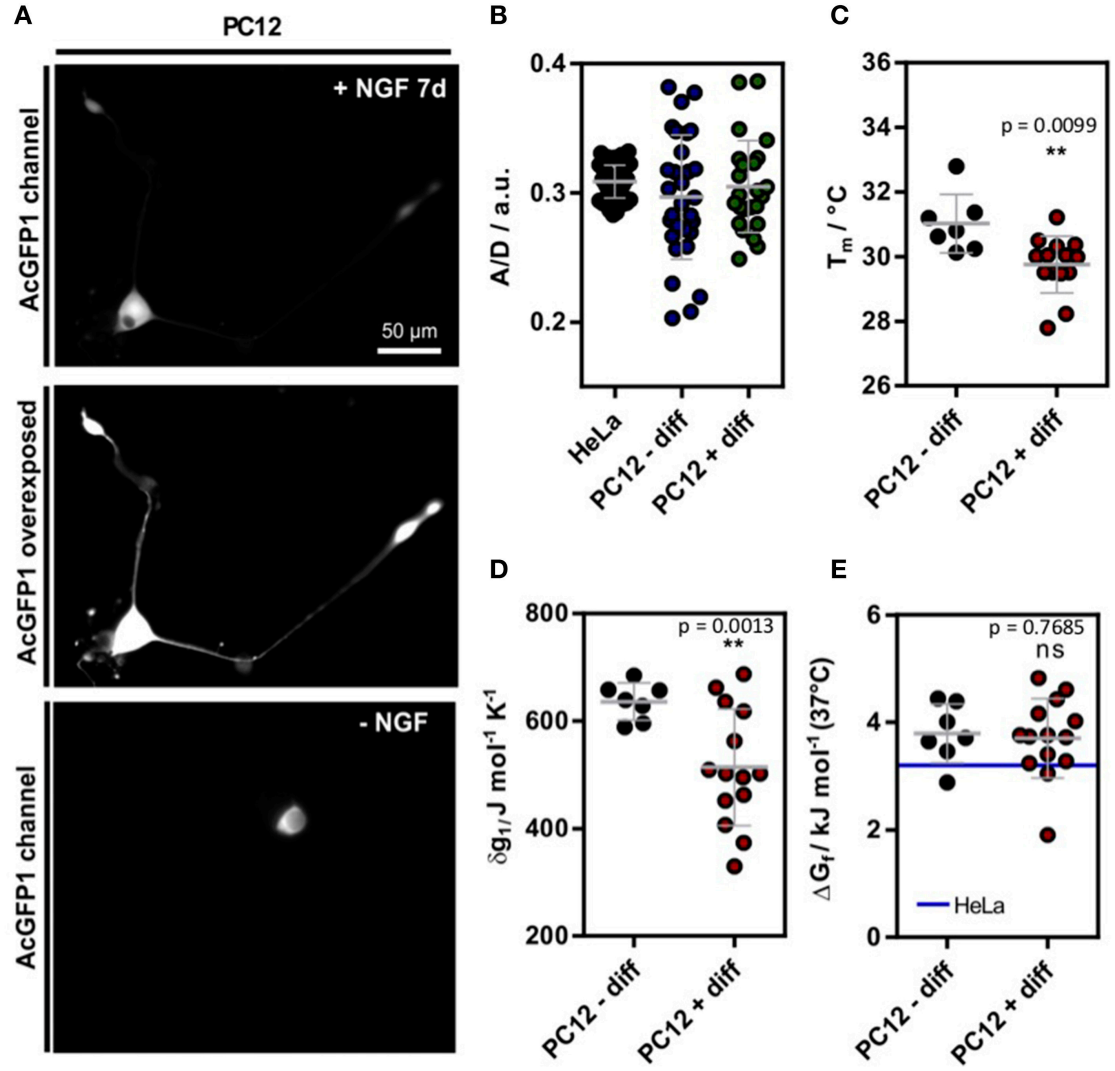
**(A)** Exemplary SOD1*_G41D_ transfected cell after 7 d incubation with 100 ng L^−1^ NGF. For better visualization, an intensity scaled version is shown. On the bottom, a control cell is shown without NGF treatment. **(B)** A/D ratio of the genetic crowding sensor (Boersma et al., 2015) in HeLa as well as PC12 cells (Figure S1) **(C)** Melting temperatures in differentiated and undifferentiated PC12 cells. **(D)** Cooperativity parameter δg1 in differentiated and undifferentiated PC12 cells. (**E)** Δ*G*_f_ in differentiated and undifferentiated PC12 cells. For comparison, the mean value of HeLa cell measurements is depicted [blue line (3.2 ± 0.5) kJ mol^−1^ (data shown from reference Gnutt et al., 2019)]. Each data point represents a single cell measurement **(B–E)**. Statistical significance was tested using either Welch's unpaired *t*-test **(B–D)** or a non-parametric Kruskal-Wallis test followed by a *post-hoc* Dunn's test for multiple comparisons **(E)**. **p < 0.01.

Next, we tested if neuronal differentiation affects intracellular protein folding stability. Therefore, we used a FRET labeled truncated superoxide dismutase 1 variant with a destabilizing mutation G41D (${\text{SOD}1}_{\text{G}41\text{D}}^{*}$) as a previously introduced folding reporter (Gnutt et al., 2019). ${\text{SOD}1}_{\text{G}41\text{D}}^{*}$ shows reversible, two-state folding properties at physiological temperature and is therefore a well-behaved protein for in-cell folding studies. Furthermore, SOD1 misfolding is implicated to play a role in familiar amyotrophic lateral sclerosis (fALS), a severe motor neuron disease (Lindberg et al., 2005). Therefore, understanding its folding in a neuronal cell model could reveal new insights into the disease-associated folding mechanism.

To study in-cell ${\text{SOD}1}_{\text{G}41\text{D}}^{*}$ folding, we used Fast Relaxation Imaging (FReI), a combination of fast fluorescence microscopy with laser induced temperature jumps (Ebbinghaus et al., 2010). Briefly, a temperature profile of successive temperature jumps (2.3°C amplitude) was used to rapidly perturb the folding equilibrium and measure folding stability and kinetics at each temperature (see Supplementary Material for details). Briefly, the relaxation kinetics were fitted using a single exponential to retrieve the respective folding amplitudes (Girdhar et al., 2011). The amplitude is proportional to the population shift and was further used to obtain the thermal melting profile of the protein which was fit using a two-state model (Figure S2) (Girdhar et al., 2011). The thermodynamic fit yielded the melting temperature T_m_ and the expansion parameter δg1, which contains enthalpic and entropic folding contributions (Girdhar et al., 2011). The standard folding free energy was linearly extrapolated by ΔG_f_ = δg1 (T – T_m_), as discussed previously (Girdhar et al., 2011).

The single-cell experiments revealed a significant reduction of T_m_ in differentiated PC12 cells (Figures 1A,C). Further, the expansion parameter δg1 decreased significantly (Figure 1D), but no change in the standard folding free energy ΔG_f_ was observed (Figure 1E). Compared to previous measurements of ${\text{SOD}1}_{\text{G}41\text{D}}^{*}$ in HeLa cells (Gnutt et al., 2019), a minor destabilization corresponding to an increase in Δ*G*_f_ was observed (blue line, Figure 1E). Further, the in-cell stabilities were significantly reduced compared to previous *in vitro* stability measurements in PBS (pH = 7.4) buffer solution (ΔG_f_ = −1.0 ± 0.2 kJ mol^−1^) (Gnutt et al., 2019). This can be explained by destabilizing quinary interactions that govern the in-cell folding stabilities for most mutants of SOD1^*^ (Gnutt et al., 2019) and is in line with previous studies of in-cell folding stabilities of the proteins VlsE and GB1 (Guzman et al., 2014;Monteith and Pielak, 2014).

Intriguingly, although crowding remained constant during differentiation, significant changes were found for T_m_ and δg1. This could be explained by changes in quinary interactions or via specific proteins expressed inside differentiated PC12 cells, e.g. molecular chaperones. Post-translational modifications (PTMs) of ${\text{SOD}1}_{\text{G}41\text{D}}^{*}$ could also cause these changes as SOD1 is phosphorylated and acetylated in cells (Tsang et al., 2014). Such PTMs could change the surface properties and thereby affect its net charge and interactions with other proteins (Mu et al., 2017). Changes to the proteome (and thus likely quinary interactions) are in line with proteomic studies reporting upregulation of GPR78, a hallmark gene of the ER unfolded protein response (UPR), and other UPR related proteins in NGF treated cells (Emdal et al., 2015). Molecular chaperones specifically target the unfolded states of proteins and could therefore lead to a shift of the protein folding equilibrium towards the unfolded state (Wood et al., 2018).

To test if changes in the proteome or chaperone capacity could modulate the in-cell folding stability, we induced proteostasis stress by inhibiting the 26S proteasome using MG132 (Lee and Goldberg, 1998) incubated HeLa cells. Proteasome inhibition leads to an overload of the cell with proteins and increases chaperone synthesis (Lee and Goldberg, 1998). Again, we used ${\text{SOD}1}_{\text{G}41\text{D}}^{*}$ as a model system to measure intracellular folding stability. Long-term incubation using 10 μM MG132 lead to clear morphological changes of the cells, in agreement with the sequestration of intracellular proteins (Figure 2A). We found a significant decrease in stability (increase in Δ*G*_f_, decrease of T_m_) and an increase in δg1 after 18 h incubation (Figure 2B). After 24 h of incubation, unfolding curves could only be resolved for some cells, putatively due to already misfolded and aggregated proteins. Upon further temperature increase, we found a decrease in D/A indicating ongoing aggregation of misfolded ${\text{SOD}1}_{\text{G}41\text{D}}^{*}$ protein (Figure 2C). This corresponds to temperature-jump induced aggregation which we previously observed for different aggregation-prone proteins in HeLa cells (Büning et al., 2017; Vöpel et al., 2017). Last, we used the crowding sensor to test if the morphological changes inside the cell alter the crowding fraction. Indeed, we found a decrease in the A/D-ratio (Figure 2D) showing that the decrease in ${\text{SOD}1}_{\text{G}41\text{D}}^{*}$ folding stability correlates with a decrease in crowding.

**Figure 2:**
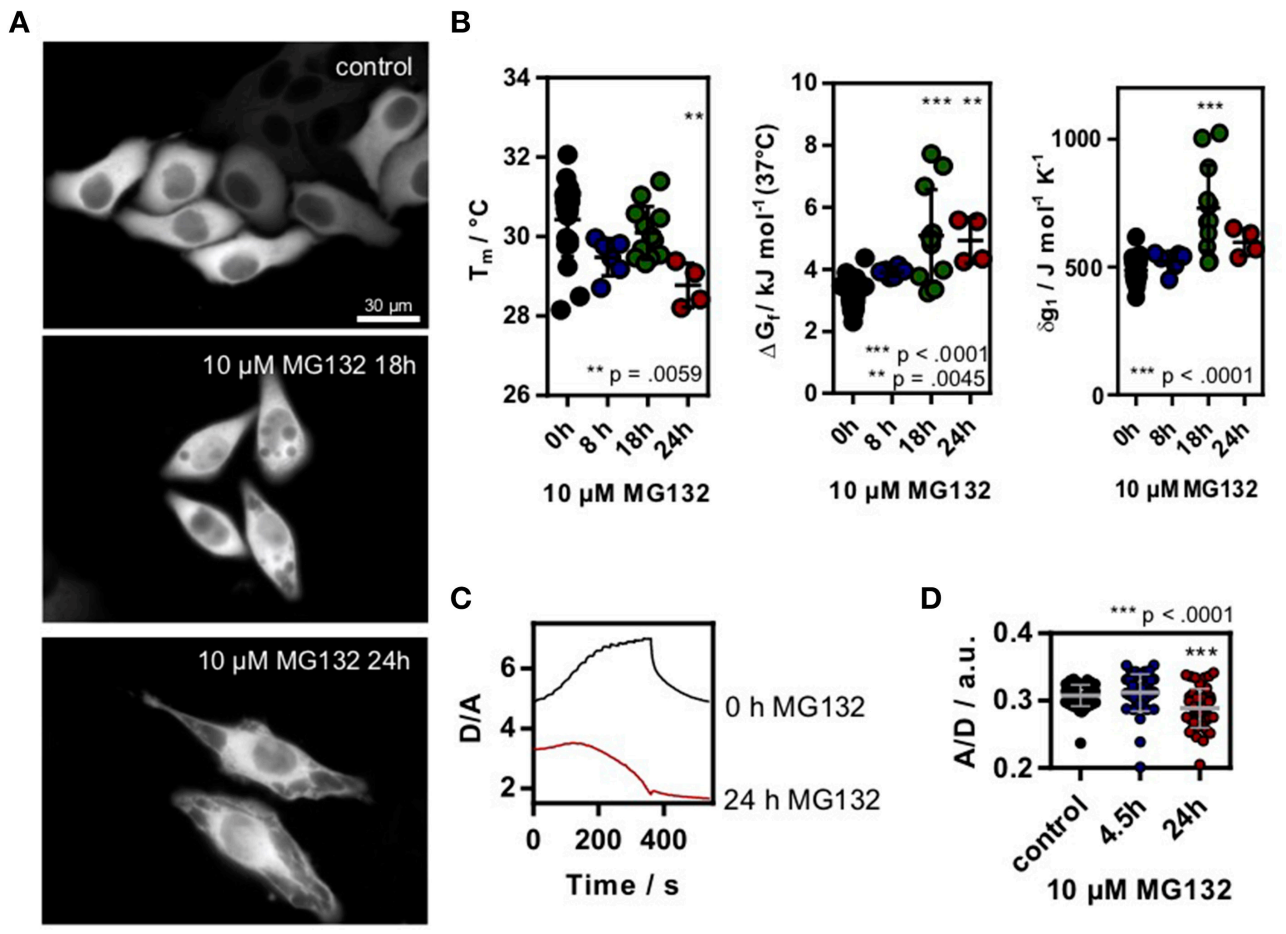
**(A)** HeLa cells transfected with SOD1*_G41D_ were treated with 10 μM MG132 for different timepoints. Morphological differences could be observed after ~18 h incubation. Scale bar 30 μm. **(B)** Melting temperature, ΔG_f_ and cooperativity parameter δg1 in MG132 treated cells. Each data point represents a single cell measurement. Error bars depict mean and s.d. **(C)** Exemplary D/A unfolding curve for a control cell and a cell treated with 10 μM MG132 for 24 h which showed a temperature triggered aggregation behavior. **(D)** A/D ratio of the genetic crowding sensor in MG132 treated cells. Each data point represents a single cell measurement **(B,D)**. Statistical significance was tested using a non-parametric Kruskal-Wallis test followed by a *post-hoc* Dunn's test for multiple comparisons. Asterisks denote differences towards the untreated control (0h). *** *p* < 0.001, ** *p* < 0.01.

In summary, we found that proteins fold differently in differentiated and stressed cells compared to unperturbed cells. Specific changes of the cellular environment could cause such effects including morphological alterations, proteasome modifications, PTMs or changes in crowding. Our results show that standard cell lines such as HeLa cells are good models to explore in-cell effects, but further experimental studies may be required when interpreting folding studies in the disease-relevant context. Such studies should also serve as a benchmark to develop and tune cytomimetic media, since such media are often required to mimic disease-like conditions. Our results show that the development of cytomimetic media needs to go beyond simple crowder solutions like BSA or Ficoll to account for various physicochemical and biological factors in cells. It becomes clear that cytomimetic media need to be individually designed for specific biomolecular systems of interest. In the protein folding case studied here, the cytomimetic medium should include a reconstituted chaperone system, membranes, crowding agents, as well as osmolytes, a tRNA pool and salts.

## Data Availability

All datasets generated for this study are included in the manuscript and/or the Supplementary Files.

## Author Contributions

DG and LS conducted experiments. DG and SE designed the research, analyzed the data and wrote the manuscript.

### Conflict of Interest Statement

The authors declare that the research was conducted in the absence of any commercial or financial relationships that could be construed as a potential conflict of interest.
