# Supplementary material for: Protein Folding Modulation in Cells Subject to Differentiation and Stress
Source: Front Mol Biosci. 2019 May 24;6:38. doi: 10.3389/fmolb.2019.00038 (PMC6544126; doi:10.3389/fmolb.2019.00038)
Supplement: Supplementary file 1 [file Table_1.docx]

**Supplementary Material**

**Material**

Plasmid generation of the SOD1 folding reporter was described previously^1^. The genetic crowding sensor containing Clover and mRuby2 as FRET pair was synthesized by Genscript based on the crowding sensor reported by Boersma *et al.^2^* All chemicals and materials were obtained by Sigma Aldrich if not otherwise declared.

**Passaging of PC12 cells**

PC12 cells were cultured using DMEM supplemented with 10% FBS, 5% HS and 1% P/S (growth medium) in poly-D-lysine coated T25 ﬂasks (0.1 mg mL^−1^). For propagation, they were grown at 37^◦^C in 5% CO2 atmosphere in a cell incubator until 70–80% conﬂuency. Commonly, cells were subcultured using 1:4 to 1:6 ratios. For experiments, cells were plated on 35 mM glass bottom dishes (WPI).

**Differentiation of PC12 cells**

Proliferation medium was exchanged to differentiation medium (DMEM with 1% HS and 1% P/S). Differentiation was then promoted using 100 ng mL^−1^ nerve growth factor (NGF). Commonly, cells differentiated for 4–7d. Differentiation medium was replaced every 2 d and new NGF added.

**Transfection of PC12 cells**

Transfection was performed using Lipofectamine 3000 (Thermo Fisher) according to the manufacturer’s manual: 2.5 µg DNA, 4 µL Lipofectamine 3000 reagent and 5 µL P3000 reagent were utilized for each transfection. Cells were transfected 2d after plating.

**HeLa cell culture & transfection**

HeLa cells were grown in standard T25 flasks using DMEM supplemented with 10% FBS, 1% P/S. Cells were subcultured at ratios of 1:4-1:6. HeLa cells were transfected using Lipofectamine 3000 according to the manufacturer’s protocol.

**Experiment preparation**

Cells were plated on poly-D-lysine coated 35 mm glass bottom dishes (WPI). For temperature jump measurements, cells were placed in a chamber of well-defined height to ensure a constant temperature profile between measurements. A sample chamber was constructed using a #1 coverslip (Menzel), a 120 µm thick spacer (Grace Bio-Labs SecureSealTM, Sigma-Aldrich) and the WPI glass bottom dish. Therefore, the coverslip was ﬁrst cleaned using EtOH (100%). The double-sided spacer was attached to the coverslip. Cells were washed twice carefully using DPBS. A drop of 31 µL L15/30% FBS was placed on the glass coverslip. After aseptically removing of DPBS, the glass bottom dish was placed inverted on the coverslip. To ensure reproducibility, sample chambers containing air bubbles were not used for further experiments.

**FReI measurements and data evaluation.**

Measurements were performed as previously described^1^. Briefly, Fast Relaxation Imaging (FReI) combines rapid temperature jumps with fluorescence microscopy, as described elsewhere^3^. An IR diode laser is used to incrementally heat the sample by 2.3 °C every 25 s from 23 °C to the maximal temperature (12 to 16 jumps for each measurement), yielding relaxation kinetics and equilibrium conformation at each temperature. Temperature jumps were calibrated using the temperature sensitive dye rhodamine B as described earlier^4,5^. Förster resonance energy transfer was measured between an AcGFP1 donor and a mCherry acceptor. The fluorescence donor was excited by using 470 nm LED light at constant exposure for each sample. Emission intensities of the AcGFP1-donor (D) (497 - 527 nm) and the mCherry-acceptor (A) (581 - 679 nm) were separated using a dichroic mirror und imaged simultaneously by two CCD cameras at a frame rate of 5 frames per second. The data was evaluated using ImageJ (US NIH) and in-house developed Matlab code. The intensities were extracted for every cell separately by first using intensity thresholding of D intensities to separate the cytosol from the nuclear region. Only cytosolic intensities were used for further analysis to prevent any skewing of the data by nuclear regions, e.g. diffusion of protein into and out of the nucleus. Further, extraction of intensities from peripheral neurite areas was not possible due to limitations in resolution and partial movements of the neurites throughout the temperature jump.

To obtain thermodynamic parameters, we used the “Better thermodynamics from kinetics” method described previously^6^. For each temperature jump, the $D-\alpha A$values were calculated and fitted by a single exponential function. The amplitudes $D-\alpha A$were plotted against the temperature of the individual jumps (Figure S2). The resulting data was fitted to equation (1) using a two-state approximation yielding δg_1_ and T_m_.

$D-\alpha A \left( T \right)=\frac{-\delta g_{1}\Delta T\cdot T_{m}}{R(T-\Delta T/2)^{2}}\cdot\left( A_{0}+m_{a}\cdot\left( T-T_{m} \right) \right)\cdot\frac{e^{-\delta g_{1}\left( T-\frac{\Delta T}{2}-T_{m} \right)\cdot\left( R\left( T-\frac{\Delta T}{2} \right) \right)^{-1}}}{\left( 1+e^{-\delta g_{1}\left( T-\frac{\Delta T}{2}-T_{m} \right)\cdot\left( R\left( T-\frac{\Delta T}{2} \right) \right)^{-1}} \right)^{2}}$ (1)

ΔG_f_ was calculated using a linear Taylor expansion ΔG_f_ = δg1 (T – Tm)^7^.

**References**

1. Gnutt, D. et al. Stability Effect of Quinary Interactions Reversed by Single Point Mutations. *Journal of the American Chemical Society* (2019).

2. Boersma, A.J., Zuhorn, I.S. & Poolman, B. A sensor for quantification of macromolecular crowding in living cells. *Nat Methods* **12**, 227-9, 1 p following 229 (2015).

3. Ebbinghaus, S., Dhar, A., McDonald, J. & Gruebele, M. Protein folding stability and dynamics imaged in a living cell. *Nature methods* **7**, 319-323 (2010).

4. Vopel, T. et al. Infrared laser triggered release of bioactive compounds from single hard shell microcapsules. *Chem Commun (Camb)* **51**, 6913-6 (2015).

5. Gao, M. et al. RNA Hairpin Folding in the Crowded Cell. *Angew Chem Int Ed Engl* **55**, 3224-8 (2016).

6. Girdhar, K., Scott, G., Chemla, Y.R. & Gruebele, M. Better biomolecule thermodynamics from kinetics. *J Chem Phys* **135**, 015102 (2011).

7. Dhar, A. et al. Protein stability and folding kinetics in the nucleus and endoplasmic reticulum of eucaryotic cells. *Biophysical journal* **101**, 421-430 (2011).

**
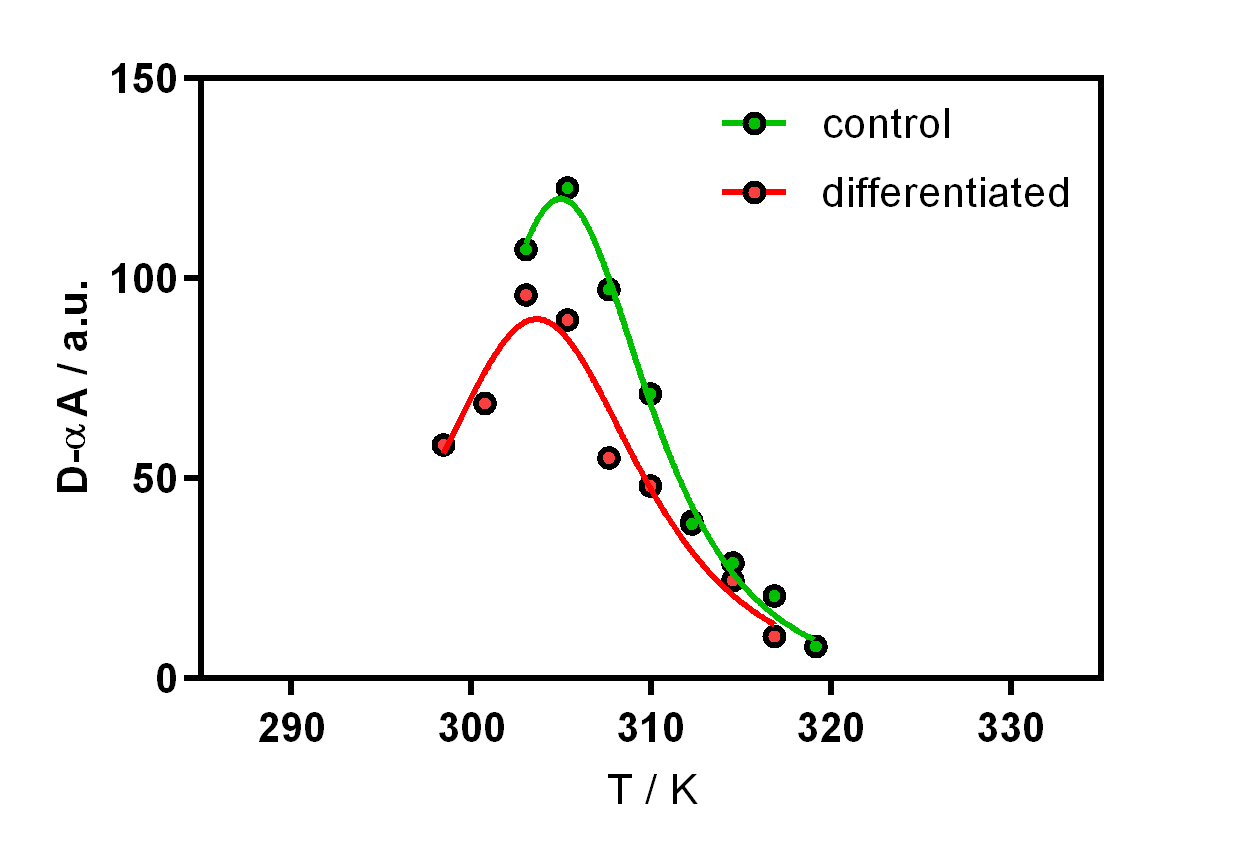

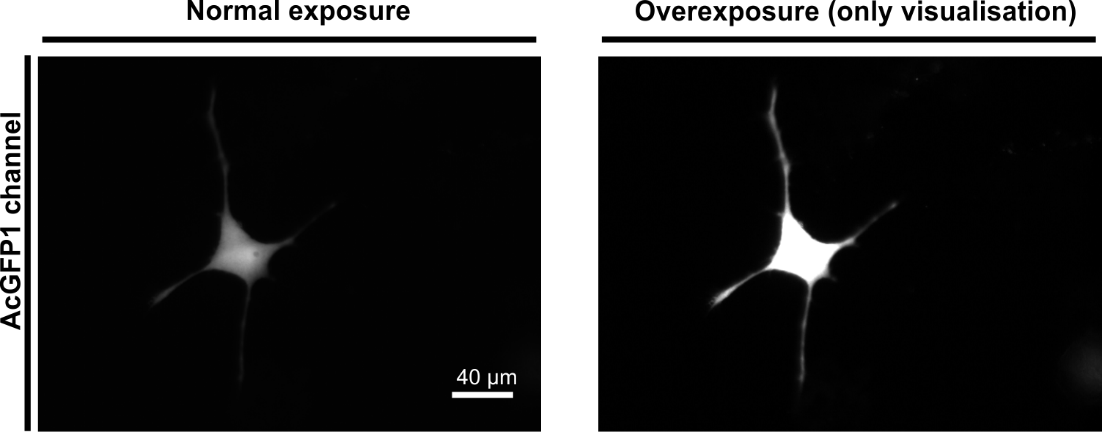
Supplementary Figures**

**Figure S1** PC12 cells transfected with the genetic crowding sensor after 7 d incubation with 100 ng L^-1^ NGF. For better visualization, an intensity scaled version is shown. Scale bar represents 40 µm.

**Figure S2** Exemplary curves of a single undifferentiated and differentiated PC12 cell to visualize the thermodynamic fitting procedure. Briefly, the kinetic amplitude is plotted against each temperature and fit using equation (1). The maximum represents the melting temperature T_m_.
